# A Generic Compliance Modeling Method for Two-Axis Elliptical-Arc-Filleted Flexure Hinges

**DOI:** 10.3390/s17092154

**Published:** 2017-09-19

**Authors:** Lijian Li, Dan Zhang, Sheng Guo, Haibo Qu

**Affiliations:** 1 School of Mechanical, Electronic and Control Engineering, Beijing Jiaotong University, Beijing 100044, China; lljianzhu@126.com (L.L.); shguo@bjtu.edu.cn (S.G.); hbqu@bjtu.edu.cn (H.Q.); 2 Department of Mechanical Engineering, York University, 4700 Keele Street, Toronto, ON M3J 1P3, Canada

**Keywords:** flexure hinge, two-axis, elliptical arc filleted, compliance modeling, flexure segment

## Abstract

As a kind of important flexible joint, two-axis flexure hinges can realize in-plane and out-of-plane motions and can be used for constructing flexure-based spatial compliant mechanisms. The paper introduces a common two-axis elliptical-arc-filleted flexure hinge that is generated by two different elliptical-arc-filleted cutout profiles and that provides some new hinge types. The analytical compliance equations of both half-segments of the two-axis elliptical-arc flexure hinges are firstly formulated, and then, based on a generic compliance modeling method of a flexure serial chain, the closed-form compliance and precision matrices of two-axis elliptical-arc-filleted flexure hinges are established and validated by the finite element method. Some numerical simulations are conducted to compare the effect of different design geometric parameters on the performance of the two-axis flexure hinges.

## 1. Introduction

Flexure hinges can utilize their slender portions to produce relative motion between two adjacent rigid links in flexure-based compliant mechanism. In many high precision and micro-operation applications such as micro/nano-positioning platforms [1,2,3], displacement amplifiers [4,5,6,7], sensors [8,9,10], elliptical-vibration texturing device [11], micro-grippers, bionics, and micro-electromechanical systems [12,13,14,15], flexure hinges also play a very important role as alternative solutions to traditional rigid joints due to their positive features of not requiring assembly, compactness, zero friction and high resolution, etc.

**Figure 1:**
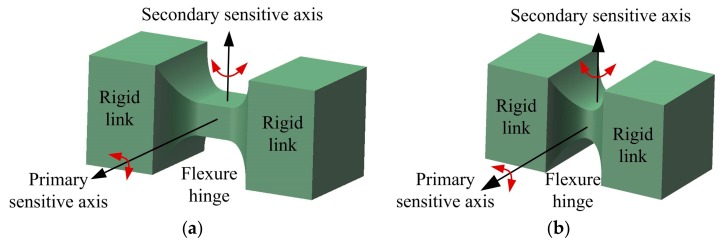
Hinge configurations: (**a**) Two-axis corner-filleted flexure hinge; (**b**) Two-axis flexure hinge.

The quasi-static responses of flexure hinges with different notch types under axial, bending, shear loading and torsion have been a research focus in the design of flexure-based compliant mechanisms. Paros and Weisbord [16] first formulated the exact and simplified compliance equations of the right circular flexure hinge. Since then, efforts to obtain precise, concise, and analytically compliance formulations of various types of flexure hinges are encouraging many researchers and scholars to explore more kinds of flexure hinges with different notch types. Normally, the notch types of flexure hinges include circular, elliptical, parabolic and hyperbolic [17,18,19,20], V-shaped [21], hybrid non-symmetric [22], and other more complex-shaped or multiple segments connected serially [23,24]. Based on the Castigliano’s second theorem and the small deformation assumptions, as well as by introducing the infinite element analysis method, the closed-form compliance and precision equations of various flexure hinges can be analytically conducted and effectively validated. However, for some special notch types, the compliance equations of flexure hinges are hard to derive analytically, or can only be obtained with numerical solutions [23] or empirical equations for some specific geometric parameters with enhanced calculation precision [25,26]. According to the capacity of rotation and the main functions, flexure hinges can be designed as single-axis, two-axis, and multiple-axis [27]. These hinge designs can be also further categorized into circular-axis and straight-axis in terms of the shapes of the central axis, or symmetric and non-symmetric whether the hinge profiles are transverse symmetry or not [28,29]. Unlike single-axis flexure hinge designs, which have only one sensitive revolute axis between two adjacent rigid links, two-axis flexure hinges have two orthogonal sensitive revolute axes at one point—see Figure 1. Figure 1a illustrates a two-axis corner-filleted hinge configuration that enables the right rigid link to produce in-plane rotation around the primary sensitive axis and out-of-plane rotation around the secondary sensitive axis with respect to the left rigid link. The in-plane or out-of-plane capacity of rotation can be evaluated by the thickness and width in the minimum cross section of two-axis flexure hinge. When disposing of the mid-segment flexure hinge with constant rectangular cross section from the two-axis corner-filleted flexure hinge, the hinge will degenerate into a general two-axis flexure hinge, as shown in Figure 1b. Meanwhile, two-axis flexure hinge can be regarded as a flexible U-joint, and possesses compact in size compared to other flexible U-joints designed in a serial or combined manner with two or multiple single-axis flexure hinges [30].

**Figure 2:**
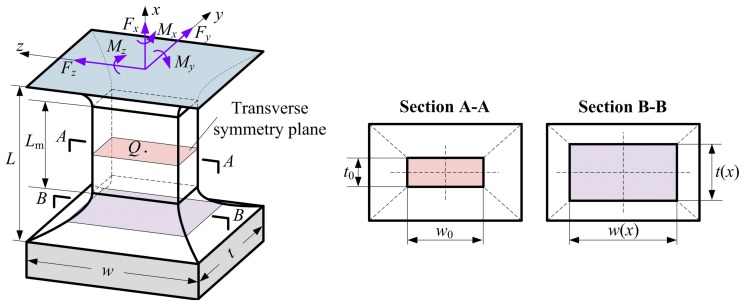
Geometric parameters and profiles of two-axis corner-filleted flexure hinge.

Several researchers have analyzed the design equations of two-axis flexure hinges. Lobontiu et al. [31] derived the closed-form compliances of two-axis flexure hinges with two non-identical parabolic profiles. The compliance equations of the double-axis elliptical flexure hinge and the bi-axial right circular flexure hinge were also formulated [32,33], respectively. Hou et al. [34] derived the precision equations of the two-axis rectangular cross-section corner-filleted flexure hinge. In order to select preferably the suitable two-axis flexure hinges for designers in the design phase, it is essential to introduce some other types of two-axis flexure hinges, especially for some most common two-axis corner-filleted flexure hinges. However, the closed-form compliance equations and precision equations for two-axis corner-filleted flexure hinges will be hard to obtain analytically by direct integration because the thickness and width of the hinges at arbitrary corner-filleted cross section are variable along the straight longitudinal axis *x*—see Figure 2, where *L* and *L*_m_ are the total length and the mid-segment length, respectively, *w*_0_ and *t*_0_ are the minimum width and the minimum thickness—respectively while—*w* and *t* are the maximum width and the maximum thickness, respectively.

**Figure 3:**
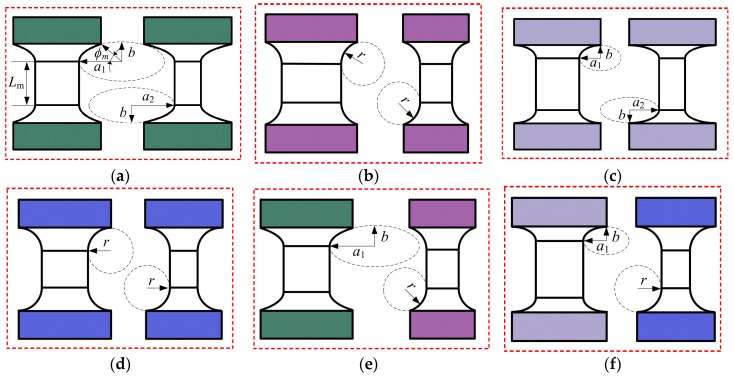
Two-axis flexure hinges with different cutout profiles: (**a**) Dual elliptical-arc-filleted (DEAF); (**b**) Dual circular-arc-filleted (DCAF, *a*_1_ = *a*_2_ = *b* = *r*); (**c**) Dual elliptical-filleted (DEF, *φ*_m_ = π/2); (**d**) Dual right-circular-filleted (DRCF, *a*_1_ = *a*_2_ = *b* = *r* and *φ*_m_ = π/2); (**e**) Hybrid elliptical-arc-filleted and circular-arc-filleted (HEACF, *a*_1_ = *b* = *r* or *a*_2_ = *b* = *r*); (**f**) Hybrid elliptical-filleted and right-circular-filleted (HERCF, *a*_1_ = *b* = *r* or *a*_2_ = *b* = *r* and *φ*_m_ = π/2).

A two-axis corner-filleted flexure hinge whose two adjacent cutout profiles are introduced and defined by two non-identical elliptical-arc-filleted, respectively, as figured in Figure 3a, where *a*_1_ and *a*_2_ indicate the major semi-axis of ellipse, *b* and *r* represent the minor semi-axis of ellipse and the radius of circle, respectively, *φ*_m_ (0 < *φ*_m_ < π/2) is the maximum semi-eccentric angle. Chen et al. [35] gave the relationships among circular, right-circular, elliptical and elliptical-arc, and the former three are subsets of the latter one. Hence, the two-axis elliptical-arc-filleted flexure hinge defined by dual elliptical-arc-filleted cutout profiles as one of the most common two-axis corner-filleted flexure hinges can be translated into other two-axis corner-filleted flexure hinges in terms of the relationships among *a*_1_, *a*_2_, *b* and *r* and the value of *φ*_m_. Figure 3b–f illustrate five two-axis corner-filleted flexure hinges, whose two different cutout profiles are defined by dual circular-arc-filleted, dual elliptical-filleted, dual right-circular-filleted, hybrid elliptical-arc-filleted and circular-arc-filleted, hybrid elliptical-filleted and right-circular-filleted, respectively. Similarly, when *L*_m_ = 0, all these two-axis corner-filleted flexure hinges above will degenerate into other six general two-axis flexure hinges.

The present work introduces a common two-axis elliptical-arc-filleted flexure hinge, and makes it piecewise and as a flexure serial chain composed of multiple flexure segments, then utilizes the compliance matrix modeling method based on the virtual theory and the superposition principle, and derives analytically the complex compliance and precision equations of two-axis elliptical-arc-filleted flexure hinge, as well as studies the sensitivity to geometric parameters.

## 2. Compliance Equations

**Figure 4:**
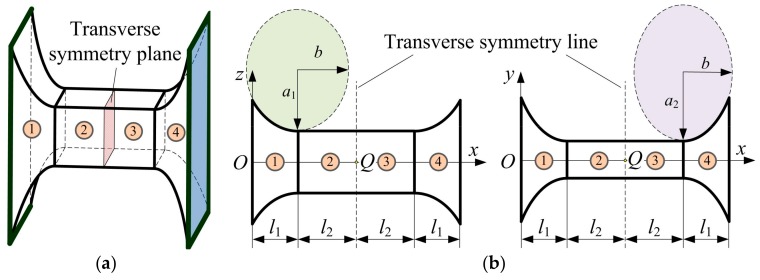
Four-segment two-axis elliptical-arc-filleted flexure hinge: (**a**) Three-dimensional view; (**b**) Geometric parameters and two non-identical cutout profiles.

A generic two-axis elliptical-arc-filleted flexure hinge with a transverse symmetry plane is shown in Figure 4a, which can be divided into four segments in terms of the cutout geometric profiles. The segments 1 and 4 are the left half-segment and right half-segment two-axis flexure hinges with elliptical-arc notches, respectively, the segments 2 and 3 are both identical constant rectangular cross-section beams. Figure 4b shows the geometric parameters, the vertical and horizontal cutout profiles through the initial center of rotation *Q*, where *l*_1_ is the length of the segments 1 and 4, and *l*_2_ is the length of the segments 2 and 3.

When a variable- thickness and width two-axis flexure hinge with elliptical-arc-filleted notches is used for flexure-based spatial compliant mechanisms, the in-plane and out-of-plane motions can be achieved, thus its compliance matrix will be a 6 × 6 square matrix. To obtain the compliance matrix of two-axis elliptical-arc-filleted flexure hinge, the closed-form compliance equation of each element of the compliance matrix needs to be derived analytically with the consideration of the shear effects.

### 2.1. Two-Axis Elliptical-Arc-Filleted Flexure Hinge

**Figure 5:**
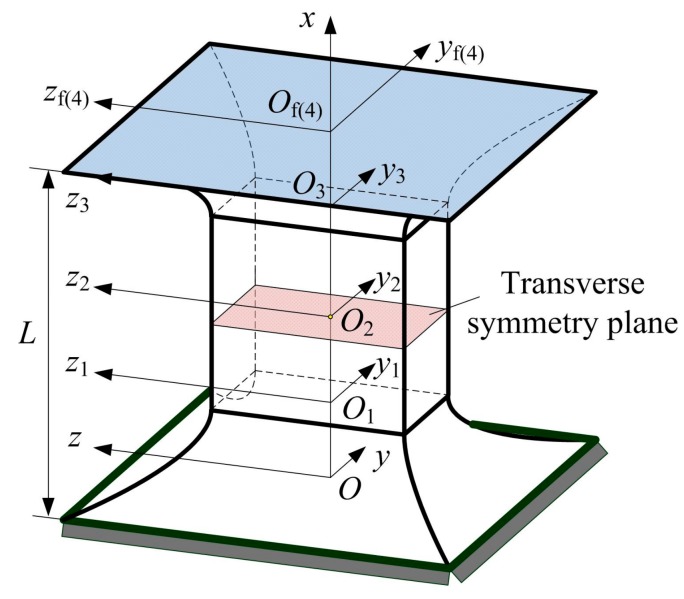
Geometric relationships among coordinate frames.

Assume that each flexure segment is a flexure member, then the two-axis elliptical-arc-filleted flexure hinge can be regarded as a flexure serial chain that is formed of four flexure members—see Figure 4a. For each fixed-free flexure member, the geometric center of the free end with a local frame can be constructed a compliance matrix with respect to the global frame established in the geometric center of the fixed end [36,37]. Furthermore, the overall compliance matrix of the multi-segment flexure hinge can be also derived through a certain operation relation depending on the local compliance matrix of all flexure members and the coordinate transformation matrix [37]. The geometric relationships among coordinate frames of two-axis elliptical-arc-filleted flexure hinge are shown in Figure 5, where the *O*-*xyz* and *O*_f_-*xy*_f_*z*_f_ are the global frame and the local frame established in the fixed end and the free end of the hinge, respectively, the *O_i_*-*xy_i_z_i_* (*i* = 1, 2, 3, 4) is the local frame established in the tip of flexure segment *i*.

Based on the virtual work theory and the superposition relationship of the deformation, the overall compliance matrix of two-axis elliptical-arc-filleted flexure hinge in the local frame *O*_f_-*xy*_f_*z*_f_ can be expressed [36,37,38]:

(1) $$C=\sum_{i=1}^{4}J_{i}C_{i}J_{i}^{T}$$

where ***C****_i_* indicates the compliance matrix of the flexure segment *i*, ***J****_i_* indicates the compliance transformation matrix, which can be given by:

(2) $$J_{i}=\left(\begin{array}{cc} R_{i} & -R_{i}S_{i}(r_{i})\\ 0_{3\times 3} & R_{i} \end{array}\right)$$

where ***R****_i_* is the orientation transformation matrix of the local frame *O_i_*-*xy_i_z_i_* with respect to the local frame *O*_f_-*xy*_f_*z*_f_, and is a unit matrix here, ***0***_3×3_ is a three order zero square matrix, ***S****_i_*(***r****_i_*) represents the skew-asymmetric operator for the position vector ***r****_i_* = [*r_ix_*, *r_iy_*, *r_iz_*]^T^ that is the origin *O*_f_ of local frame *O*_f_-*xy*_f_*z*_f_ in the local frame *O_i_*-*xy_i_z_i_*, which both can be expressed as:

(3) $$S_{i}(r_{i})=\left(\begin{array}{ccc} 0 & -r_{iz} & r_{iy}\\ r_{iz} & 0 & -r_{ix}\\ -r_{iy} & r_{ix} & 0 \end{array}\right)$$

(4) $$r_{1}={\left(\begin{array}{ccc} l_{1}+2l_{2} & 0 & 0 \end{array}\right)}^{T};r_{2}={\left(\begin{array}{ccc} l_{1}+l_{2} & 0 & 0 \end{array}\right)}^{T};r_{3}={\left(\begin{array}{ccc} l_{1} & 0 & 0 \end{array}\right)}^{T};r_{4}={\left(\begin{array}{ccc} 0 & 0 & 0 \end{array}\right)}^{T}$$

For the constant rectangular cross-section flexure beam, its compliance matrix has been widely studied and has high calculation precision, thus the compliance matrix of flexure segments 2 and 3 can be directly given as follows [28,36]:

(5) $$C_{2(3)}=\left(\begin{array}{cccccc} C_{2(3),11} & 0 & 0 & 0 & 0 & 0\\ 0 & C_{2(3),22} & 0 & 0 & 0 & C_{2(3),26}\\ 0 & 0 & C_{2(3),33} & 0 & C_{2(3),35} & 0\\ 0 & 0 & 0 & C_{2(3),44} & 0 & 0\\ 0 & 0 & C_{2(3),53} & 0 & C_{2(3),55} & 0\\ 0 & C_{2(3),62} & 0 & 0 & 0 & C_{2(3),66} \end{array}\right)$$

where each non-zero compliance element of compliance matrix ***C***_2(3)_ (the subscript “2(3)” indicates ***C***_2_ (or ***C***_3_)) can be calculated as:

(6) $$\left\{ \begin{array}{l} C_{2(3),11}=\frac{l_{2}}{EA_{2(3)}};C_{2(3),22}=\frac{l_{2}^{3}}{3EI_{z_{2(3)}}}+\frac{\alpha_{s}l_{2}}{GA_{2(3)}};C_{2(3),33}=\frac{l_{2}^{3}}{3EI_{y_{2(3)}}}+\frac{\alpha_{s}l_{2}}{GA_{2(3)}}\\ C_{2(3),26}=C_{2(3),62}=\frac{l_{2}^{2}}{2EI_{z_{2(3)}}};C_{2(3),35}=C_{2(3),53}=-\frac{l_{2}^{2}}{2EI_{y_{2(3)}}}\\ C_{2(3),44}=\frac{l_{2}}{GJ_{P_{2(3)}}};C_{2(3),55}=\frac{l_{2(3)}}{EI_{y_{2(3)}}};C_{2(3),66}=\frac{l_{2}}{EI_{z_{2(3)}}} \end{array}\right.$$

where *E* and *G* indicate the modulus of elasticity and the shear modulus of material, respectively, *A*_2(3)_ = *w*_0_*t*_0_ is the cross-section area, and *I_y_2(3)__* = $w_{0}^{3}$*t*_0_/12 and *I_z_2(3)__* = *w*_0_$t_{0}^{3}$/12 are the second moment of the cross-section area. For short beams or short flexure hinges (with a length-to width ratio of less than 5), the shear effects cannot be neglected [27], and for the rectangular cross section, the shear coefficient *α*_s_ is equal to (12 + 11*μ*)/[10(1 + μ)] according to the Timoshenko beam theory [39], where *μ* is the Poisson ratio of material, *J*_P_2(3)__ indicates the torsional moment of inertia of rectangular cross section beam, which can be expressed as [40]:

(7) $$J_{P_{2(3)}}=w_{0}t_{0}^{3}\left\{1/3-0.21(t_{0}/w_{0})[1-{\left(t_{0}/w_{0}\right)}^{4}/12]\right\}$$

It can be observed that the only compliance matrices of flexure segments 1 and 4 are unknown by substituting Equations (2)–(7) into Equation (1). Therefore, the compliance calculation of two-axis elliptical-arc-filleted flexure hinge can be transformed into the problem that solves individually the compliance matrices ***C***_1_ and ***C***_4_ of left half-segment and right half-segment two-axis flexure hinges with elliptical-arc notches.

### 2.2. Left Half-Segment Two-Axis Flexure Hinge with Elliptical-Arc Notches

**Figure 6:**
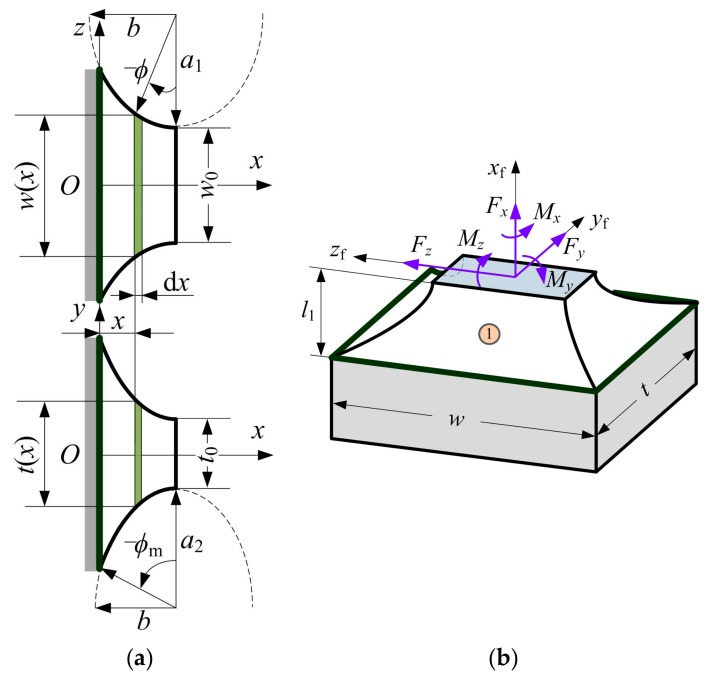
Left half-segment two-axis elliptical-arc flexure hinge: (**a**) Geometric parameters defining two non-identical symmetric elliptical-arc profiles; (**b**) Three-dimensional view.

Several assumptions are given, such as a flexure hinge can be as a six degree-of-freedom, small-deformation, fixed-free beam, and so on [35,41], and the Castigliano’s second theorem (which states that if the strain energy *U*_ε_ stored in a linear elastic body can be expressed as the function of the generalized forces *F*_P1_, *F*_P2_, …, *F*_P*N*_ acting on the elastic body, and then the first-order partial derivative of the strain energy *U*_ε_ with respect to the generalized force *F*_P*I*_ will produce a corresponding generalized displacement ∆*_I_* (*I* = 1, 2, …, *N*) along the direction of the generalized force *F*_P*I*_ [27]) is used for deriving the closed-from compliance equations of flexure segments 1 and 4. The geometric parameters and cutout profiles of the flexure segment 1 are shown in Figure 6, where *w*(*x*) and *t*(*x*) indicate the variable width in plane *xz* and the variable thickness in plane *xy* of the infinitesimal strip d*x* at position *x*, *φ* is the eccentric angle that traverses from −*φ*_m_ to 0, and also as the integral variable.

As shown in Figure 6a, the width *w*(*x*) in plane *xz* and the thickness *t*(*x*) in plane *xy* of the infinitesimal strip d*x* at position *x* can be expressed as:

(8) $$\{\begin{array}{c} w(x)=2z(x)=2a_{1}\left(1+\xi_{1}-\sqrt{1-\frac{{\left(x-l_{1}\right)}^{2}}{b^{2}}}\right)\\ t(x)=2y(x)=2a_{2}\left(1+\xi_{2}-\sqrt{1-\frac{{\left(x-l_{1}\right)}^{2}}{b^{2}}}\right) \end{array}$$

where *ξ*_1_ = *w*_0_/2*a*_1_, *ξ*_2_ = *t*_0_/2*a*_2_.

Assume that *ξ*_1_ ≠ *ξ*_2_, and by introducing the eccentric angle *φ*, we have:

(9) $$x=b\sin\phi +l_{1}$$

Differentiating Equation (9) yields:

(10) dx=bcosϕdϕ

Thus Equation (8) can be rearranged as:

(11) $$\{\begin{array}{c} w(\phi )=2a_{1}\left(1+\xi_{1}-\cos\phi \right)\\ t(\phi )=2a_{2}\left(1+\xi_{2}-\cos\phi \right) \end{array}$$

where *φ* ∈ [−*φ*_m_,0], and *w*(*φ*), *t*(*φ*) are even functions, the maximum eccentric angle *φ*_m_ = arcsin(*l*_1_/*b*).

Under the small deformation assumptions, the deformation vector ***X*** = [*u_x_*, *u_y_*, *u_z_*, α*_x_*, α*_y_*, α*_z_*]^T^ and the loading vector ***F*** = [*F_x_*, *F_y_*, *F_z_*, *M_x_*, *M_y_*, *M_z_*]^T^ applied at the free end of the flexure segment 1 satisfies the following relationship:

(12) $$X=C_{1}F$$

where ***C***_1_ is the compliance matrix of the flexure segment 1, each of its non-zero element can be determined by using the universal analytical method proposed by Ivanov and Corves [42] based on the Castigliano’s second theorem and by introducing the torsional compliance equation proposed by Chen and Howell [43] as follows:

(13) $$C_{1,11}=u_{x}/F_{x}=N_{1,1}/E$$

(14) $$C_{1,22}=u_{y}/F_{y}=12(l_{1}^{2}N_{1,2}-2l_{1}N_{1,3}+N_{1,4})/E+\alpha_{s}N_{1,1}/G$$

(15) $$C_{1,33}=u_{z}/F_{z}=12(l_{1}^{2}N_{1,5}-2l_{1}N_{1,6}+N_{1,7})/E+\alpha_{s}N_{1,1}/G$$

(16) $$C_{1,26}=u_{y}/M_{z}=C_{1,62}=\alpha_{z}/F_{y}=12(l_{1}N_{1,2}-N_{1,3})/E$$

(17) $$C_{1,35}=u_{z}/M_{y}=C_{1,53}=\alpha_{y}/F_{z}=12(N_{1,6}-l_{1}N_{1,5})/E$$

(18) $$C_{1,44}=\alpha_{x}/M_{x}=7(w_{0}^{2}+2.609w_{0}t_{0}+t_{0}^{2})(N_{1,2}+N_{1,5})/[2G(1.17w_{0}^{2}+2.191w_{0}t_{0}+1.17t_{0}^{2})]$$

(19) $$C_{1,55}=\alpha_{y}/M_{y}=12N_{1,5}/E$$

(20) $$C_{1,66}=\alpha_{z}/M_{z}=12N_{1,2}/E$$

where the newly-introduced integral factors from *N*_1,1_ to *N*_1,7_ (the subscript “1” denotes these integral factors are related to the flexure segment 1) can be expressed as:

(21) $$\left\{ \begin{array}{l} N_{1,1}=\int_{0}^{l_{1}}\frac{dx}{w(x)t(x)};N_{1,2}=\int_{0}^{l_{1}}\frac{dx}{w(x)t^{3}(x)};N_{1,3}=\int_{0}^{l_{1}}\frac{xdx}{w(x)t^{3}(x)};N_{1,4}=\int_{0}^{l_{1}}\frac{x^{2}dx}{w(x)t^{3}(x)}\\ N_{1,5}=\int_{0}^{l_{1}}\frac{dx}{w^{3}(x)t(x)};N_{1,6}=\int_{0}^{l_{1}}\frac{xdx}{w^{3}(x)t(x)};N_{1,7}=\int_{0}^{l_{1}}\frac{x^{2}dx}{w^{3}(x)t(x)} \end{array}\right.$$

### 2.3. Right Half-Segment Two-Axis Flexure Hinge with Elliptical-Arc Notches

**Figure 7:**
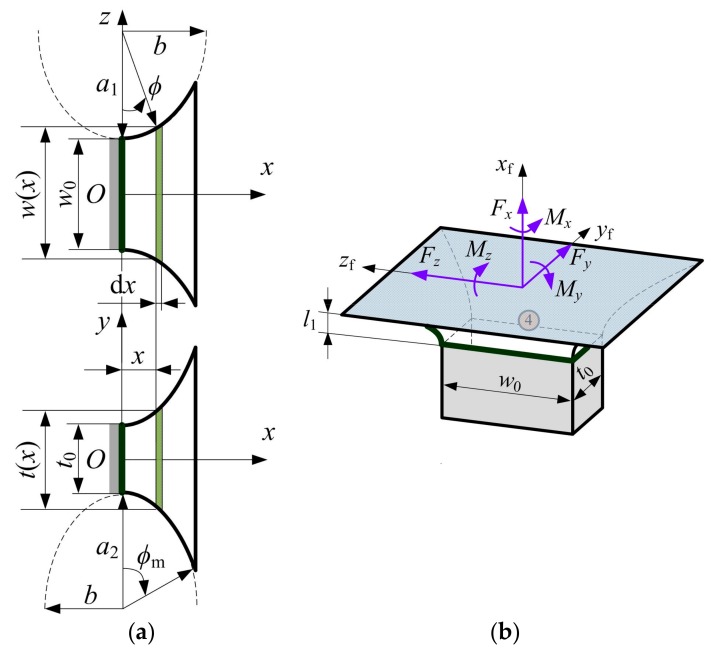
Right half-segment two-axis elliptical-arc flexure hinge: (**a**) Geometric parameters defining two non-identical symmetric elliptical-arc profiles; (**b**) Three-dimensional view.

The geometric parameters and cutout profiles of the flexure segment 4 are sketched in Figure 7. Some identical parameters defined in Figure 6 can apply to the Figure 7. As shown in Figure 7a, the width *w*(*x*) in plane *xz* and the thickness *t*(*x*) in plane *xy* of the infinitesimal strip d*x* at position *x* can be expressed as:

(22) $$\{\begin{array}{c} w(x)=2z(x)=2a_{1}\left(1+\xi_{1}-\sqrt{1-\frac{x^{2}}{b^{2}}}\right)\\ t(x)=2y(x)=2a_{2}\left(1+\xi_{2}-\sqrt{1-\frac{x^{2}}{b^{2}}}\right) \end{array}$$

Similarly, we have:

(23) x=bsinϕ

Differentiating Equation (23) yields:

(24) dx=bcosϕdϕ

Thus Equation (22) can be rearranged as:

(25) $$\{\begin{array}{c} w(\phi )=2a_{1}\left(1+\xi_{1}-\cos\phi \right)\\ t(\phi )=2a_{2}\left(1+\xi_{2}-\cos\phi \right) \end{array}$$

where *φ* ∈ [0, *φ*_m_].

For the flexure segment 4, the linear deformation *u_x_* produced by pure force *F_x_*, and the angular deformations *α_x_*, *α_y_* and *α_z_* produced by pure moment *M_x_*, *M_y_* and *M_z_*, respectively, are equal to the corresponding linear and angular deformations of the flexure segment 1 under the same conditions, namely:

(26) $$C_{4,11}=C_{1,11};C_{4,44}=C_{1,44};C_{4,55}=C_{1,55};C_{4,66}=C_{1,66}$$

Thus, we have:

(27) $$N_{4,1}=N_{1,1};N_{4,2}=N_{1,2};N_{4,5}=N_{1,5}$$

However, the other compliance elements *C*_4,22_, *C*_4,33_, *C*_4,26_ or *C*_4,62_, *C*_4,35_ or *C*_4,53_ need to be calculated individually, which can be expressed as:

(28) $$C_{4,22}=u_{y}/F_{y}=12(l_{1}^{2}N_{1,2}-2l_{1}N_{4,3}+N_{4,4})/E+\alpha_{s}N_{1,1}/G$$

(29) $$C_{4,33}=u_{z}/F_{z}=12(l_{1}^{2}N_{1,5}-2l_{1}N_{4,6}+N_{4,7})/E+\alpha_{s}N_{1,1}/G$$

(30) $$C_{4,26}=u_{y}/M_{z}=C_{4,62}=\alpha_{z}/F_{y}=12(l_{1}N_{1,2}-N_{4,3})/E$$

(31) $$C_{4,35}=u_{z}/M_{y}=C_{4,53}=\alpha_{y}/F_{z}=12(N_{4,6}-l_{1}N_{1,5})/E$$

where the newly-introduced integral factors from *N*_4,3_ to *N*_4,7_ (the subscript “4” denotes these integral factors are related to the flexure segment 4) can be expressed as:

(32) $$N_{4,3}=\int_{0}^{l_{1}}\frac{xdx}{w(x)t^{3}(x)};N_{4,4}=\int_{0}^{l_{1}}\frac{x^{2}dx}{w(x)t^{3}(x)};N_{4,6}=\int_{0}^{l_{1}}\frac{xdx}{w^{3}(x)t(x)};N_{4,7}=\int_{0}^{l_{1}}\frac{x^{2}dx}{w^{3}(x)t(x)}$$

### 2.4. Precision of Rotation

**Figure 8:**
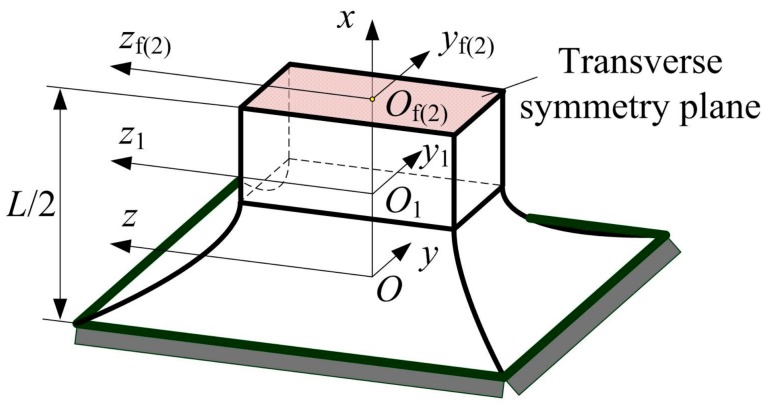
Geometric relationships among coordinate frames.

Unlike rigid revolute joints that have a fixed center of rotation, the geometric center of rotation of flexure hinges will change due to the elastic deformations. This will decrease or affect the motion accuracy of flexure-based compliant mechanisms. For symmetric two-axis flexure hinges, its linear compliance elements at the midpoint *Q* in the transverse symmetry plane are the key parameters for determining the offset of the rotational center and evaluating the precision of rotation of flexure hinges [19,20,21,31]. Therefore, the compliance matrix of the left half-segment two-axis elliptical-arc-filleted flexure hinge composed of flexure segments 1 and 2 needs to be solved. The coordinate frames established in the tips of flexure segments and their geometric relationships are in Figure 8.

Similar to Equation (1), the compliance matrix of the left half-segment two-axis elliptical-arc-filleted flexure hinge at the flexure midpoint *Q* can be expressed as:

(33) $$C_{Q}=J_{Q}C_{1}J_{Q}^{T}+C_{2}$$

where ***J****_Q_* indicates the compliance transformation matrix, which can be given by:

(34) $$J_{Q}=\left(\begin{array}{cc} R_{Q} & -R_{Q}S(r_{Q})\\ 0_{3\times 3} & R_{Q} \end{array}\right)$$

where ***R****_Q_* is a unit matrix here, the position vector ***r****_Q_* can be expressed as:

(35) $$r_{Q}={\left(\begin{array}{ccc} l_{2} & 0 & 0 \end{array}\right)}^{T}$$

Substituting Equations (34) and (35) into Equation (33), and then rearranging the Equation (33), the precision matrix of two-axis elliptical-arc-filleted flexure hinge can be given by:

(36) $$C_{P}=\left(\begin{array}{cccccc} C_{Q,11} & 0 & 0 & 0 & 0 & 0\\ 0 & C_{Q,22} & 0 & 0 & 0 & C_{Q,26}\\ 0 & 0 & C_{Q,33} & 0 & C_{Q,35} & 0 \end{array}\right)$$

where each element in the precision matrix is called a precision factor, and all precision factors of the precision matrix ***C***_P_ can be expressed explicitly as:

(37) $$\left\{ \begin{array}{l} C_{Q,11}=C_{1,11}+C_{2,11}\\ C_{Q,22}=C_{1,22}\;+\;C_{2,22}\;+\;2l_{2}C_{1,26}\;+\;l_{2}^{2}C_{1,66}\\ C_{Q,33}=C_{1,33}\;+\;C_{2,33}-2l_{2}\;C_{1,35}+\;l_{2}^{2}C_{1,55}\\ C_{Q,26}=C_{1,26}\;+\;C_{2,26}\;+\;l_{2}C_{1,66}\\ C_{Q,35}=C_{1,35}\;+\;C_{2,35}-l_{2}C_{1,55} \end{array}\right.$$

When the length *l*_2_ of flexure segment 2 is equal to zero, these precision factors in Equation (37) will be equal to the corresponding compliance elements of the left half-segment two-axis flexure hinge with elliptical-arc notches. However, when joining the serially-connected flexure segment 2, we will have to consider the effect of the compliance elements *C*_1,55_ and *C*_1,66_ on other precision factors of the precision matrix except the precision factor *C_Q_*_,11_.

## 3. Integrals in the Compliance Equations

Now we start to calculate these important and complex integrals given in Equations (21) and (32) in this section.

### 3.1. Integrals Simplification

Substituting Equations (9)–(11) into Equation (21), yields:

(38) $$N_{1,1}=\frac{b}{4a_{1}a_{2}}\int_{0}^{\phi_{m}}\frac{\cos\phi d\phi }{\left(1+\xi_{1}-\cos\phi \right)\left(1+\xi_{2}-\cos\phi \right)}=\frac{b}{4a_{1}a_{2}}I_{1}\left(\xi_{1},\xi_{2}\right)$$

(39) $$N_{1,2}=\frac{b}{16a_{1}a_{2}^{3}}\int_{0}^{\phi_{m}}\frac{\cos\phi d\phi }{\left(1+\xi_{1}-\cos\phi \right){\left(1+\xi_{2}-\cos\phi \right)}^{3}}=\frac{b}{16a_{1}a_{2}^{3}}I_{2}\left(\xi_{1},\xi_{2}\right)$$

(40) $$N_{1,3}=\frac{b}{16a_{1}a_{2}^{3}}\int_{-\phi_{m}}^{0}\frac{\left(b\sin\phi +l_{1}\right)\cos\phi d\phi }{\left(1+\xi_{1}-\cos\phi \right){\left(1+\xi_{2}-\cos\phi \right)}^{3}}=\frac{b}{16a_{1}a_{2}^{3}}\left(l_{1}I_{2}\left(\xi_{1},\xi_{2}\right)-bI_{3}\left(\xi_{1},\xi_{2}\right)\right)$$

(41) $$N_{1,4}=\frac{b}{16a_{1}a_{2}^{3}}\int_{-\phi_{m}}^{0}\frac{{\left(b\sin\phi +l_{1}\right)}^{2}\cos\phi d\phi }{\left(1+\xi_{1}-\cos\phi \right){\left(1+\xi_{2}-\cos\phi \right)}^{3}}=\frac{b}{16a_{1}a_{2}^{3}}\left(\begin{array}{l} \left(b^{2}+l_{1}^{2}\right)I_{2}\left(\xi_{1},\xi_{2}\right)-\\ 2bl_{1}I_{3}\left(\xi_{1},\xi_{2}\right)-b^{2}I_{4}\left(\xi_{1},\xi_{2}\right) \end{array}\right)$$

(42) $$N_{1,5}=\frac{b}{16a_{1}^{3}a_{2}}\int_{0}^{\phi_{m}}\frac{\cos\phi d\phi }{\left(1+\xi_{2}-\cos\phi \right){\left(1+\xi_{1}-\cos\phi \right)}^{3}}=\frac{b}{16a_{1}^{3}a_{2}}I_{2}\left(\xi_{2},\xi_{1}\right)$$

(43) $$N_{1,6}=\frac{b}{16a_{1}^{3}a_{2}}\int_{-\phi_{m}}^{0}\frac{\left(b\sin\phi +l_{1}\right)\cos\phi d\phi }{\left(1+\xi_{2}-\cos\phi \right){\left(1+\xi_{1}-\cos\phi \right)}^{3}}=\frac{b}{16a_{1}^{3}a_{2}}\left(l_{1}I_{2}\left(\xi_{2},\xi_{1}\right)-bI_{3}\left(\xi_{2},\xi_{1}\right)\right)$$

(44) $$N_{1,7}=\frac{b}{16a_{1}^{3}a_{2}}\int_{-\phi_{m}}^{0}\frac{{\left(b\sin\phi +l_{1}\right)}^{2}\cos\phi d\phi }{\left(1+\xi_{2}-\cos\phi \right){\left(1+\xi_{1}-\cos\phi \right)}^{3}}=\frac{b}{16a_{1}^{3}a_{2}}\left(\begin{array}{l} \left(b^{2}+l_{1}^{2}\right)I_{2}\left(\xi_{2},\xi_{1}\right)-\\ 2bl_{1}I_{3}\left(\xi_{2},\xi_{1}\right)-b^{2}I_{4}\left(\xi_{2},\xi_{1}\right) \end{array}\right)$$

where *I*_1_(*ξ*_1_, *ξ*_2_), *I_m_*(*ξ*_1_, *ξ*_2_) and *I_m_*(*ξ*_2_, *ξ*_1_) (*m* = 2, 3, 4) are new definite integrals about the eccentric angle *φ*, of which the non-dimensional parameters *ξ*_1_ and *ξ*_2_ of the corresponding *I_m_*(*ξ*_1_, *ξ*_2_) just need to switch each other when calculating these integrals from *I*_2_(*ξ*_2_, *ξ*_1_) to *I*_4_(*ξ*_2_, *ξ*_1_), thus we only need to derive these definite integrals from *I*_1_(*ξ*_1_, *ξ*_2_) to *I*_4_(*ξ*_1_, *ξ*_2_) individually.

Substituting Equations (23)–(25) into Equation (32), we have:

(45) $$N_{4,3}=\frac{b^{2}}{16a_{1}a_{2}^{3}}\int_{0}^{\phi_{m}}\frac{\sin\phi \cos\phi d\phi }{\left(1+\xi_{1}-\cos\phi \right){\left(1+\xi_{2}-\cos\phi \right)}^{3}}=\frac{b^{2}}{16a_{1}a_{2}^{3}}I_{3}\left(\xi_{1},\xi_{2}\right)$$

(46) $$N_{4,4}=\frac{b^{3}}{16a_{1}a_{2}^{3}}\int_{0}^{\phi_{m}}\frac{\sin^{2}\phi \cos\phi d\phi }{\left(1+\xi_{1}-\cos\phi \right){\left(1+\xi_{2}-\cos\phi \right)}^{3}}=\frac{b^{3}}{16a_{1}a_{2}^{3}}\left(I_{2}\left(\xi_{1},\xi_{2}\right)-I_{4}\left(\xi_{1},\xi_{2}\right)\right)$$

(47) $$N_{4,6}=\frac{b^{2}}{16a_{1}^{3}a_{2}}\int_{0}^{\phi_{m}}\frac{\sin\phi \cos\phi d\phi }{\left(1+\xi_{2}-\cos\phi \right){\left(1+\xi_{1}-\cos\phi \right)}^{3}}=\frac{b^{2}}{16a_{1}^{3}a_{2}}I_{3}\left(\xi_{2},\xi_{1}\right)$$

(48) $$N_{4,7}=\frac{b^{3}}{16a_{1}^{3}a_{2}}\int_{0}^{\phi_{m}}\frac{\sin^{2}\phi \cos\phi d\phi }{\left(1+\xi_{2}-\cos\phi \right){\left(1+\xi_{1}-\cos\phi \right)}^{3}}=\frac{b^{3}}{16a_{1}^{3}a_{2}}\left(I_{2}\left(\xi_{2},\xi_{1}\right)-I_{4}\left(\xi_{2},\xi_{1}\right)\right)$$

### 3.2. Integrals Calculation

These important definite integrals from *I*_1_(*ξ*_1_, *ξ*_2_) to *I*_4_(*ξ*_1_, *ξ*_2_) defined in Equations (38)–(41) can be rearranged as:

(49) $$I_{1}\left(\xi_{1},\xi_{2}\right)=\int_{0}^{\phi_{m}}\frac{\cos\phi d\phi }{\left(1+\xi_{1}-\cos\phi \right)\left(1+\xi_{2}-\cos\phi \right)}=A_{1}g_{1}\left(\xi_{1}\right)+B_{1}g_{1}\left(\xi_{2}\right)$$

(50) $$I_{2}\left(\xi_{1},\xi_{2}\right)=\int_{0}^{\phi_{m}}\frac{\cos\phi d\phi }{\left(1+\xi_{1}-\cos\phi \right){\left(1+\xi_{2}-\cos\phi \right)}^{3}}=A_{2}g_{1}\left(\xi_{1}\right)+B_{2}g_{2}\left(\xi_{2}\right)+C_{2}g_{3}\left(\xi_{2}\right)+D_{2}g_{4}\left(\xi_{2}\right)$$

(51) $$I_{3}\left(\xi_{1},\xi_{2}\right)=\int_{0}^{\phi_{m}}\frac{\sin\phi \cos\phi d\phi }{\left(1+\xi_{1}-\cos\phi \right){\left(1+\xi_{2}-\cos\phi \right)}^{3}}=-\left(A_{2}g_{5}\left(\xi_{1}\right)+B_{2}g_{6}\left(\xi_{2}\right)+C_{2}g_{7}\left(\xi_{2}\right)+D_{2}g_{8}\left(\xi_{2}\right)\right)$$

(52) $$I_{4}\left(\xi_{1},\xi_{2}\right)=\int_{0}^{\phi_{m}}\frac{\cos^{3}\phi d\phi }{\left(1+\xi_{1}-\cos\phi \right){\left(1+\xi_{2}-\cos\phi \right)}^{3}}=A_{3}g_{1}\left(\xi_{1}\right)+B_{3}g_{2}\left(\xi_{2}\right)+C_{3}g_{3}\left(\xi_{2}\right)+D_{3}g_{4}\left(\xi_{2}\right)$$

where the coefficients from *A_k_* to *D_k_* (*k* = 1, 2, 3) are related to *ξ*_1_ and *ξ*_2_, *g*_1_(*ξ*_1_), *g*_5_(*ξ*_1_) and *g_n_*(*ξ*_2_) (*n* = 1, 2, …, 8) are the definite integrals about the eccentric angle *φ*, the parameters *ξ*_1_ or *ξ*_2_ need to switch each other when calculating *g*_1_(*ξ*_2_), *g*_5_(*ξ*_2_) or *g_n_*(*ξ*_1_) used for calculating these integrals from *I*_2_(*ξ*_2_, *ξ*_1_) to *I*_4_(*ξ*_2_, *ξ*_1_). These coefficients from *A_k_* to *D_k_* and integrals *g*_1_(*ξ*_1_), *g*_5_(*ξ*_1_) and *g_n_*(*ξ*_2_) can be calculated as:

(53) $$A_{1}=-\frac{1+\xi_{1}}{\xi_{1}-\xi_{2}};B_{1}=\frac{1+\xi_{2}}{\xi_{1}-\xi_{2}}$$

(54) $$A_{2}=-\frac{1+\xi_{1}}{{\left(\xi_{1}-\xi_{2}\right)}^{3}};B_{2}={\left(\frac{1+\xi_{2}}{\xi_{1}-\xi_{2}}\right)}^{3};C_{2}=\frac{\left(1+\xi_{1}\right)\left(\xi_{1}-3\xi_{2}-2\right)}{{\left(\xi_{1}-\xi_{2}\right)}^{3}};D_{2}=\frac{1+\xi_{1}}{{\left(\xi_{1}-\xi_{2}\right)}^{3}},$$

(55) $$\left\{ \begin{array}{l} A_{3}=-{\left(\frac{1+\xi_{1}}{\xi_{1}-\xi_{2}}\right)}^{3};B_{3}=\frac{{\left(1+\xi_{1}\right)}^{2}{\left(1+\xi_{2}\right)}^{3}}{{\left(\xi_{1}-\xi_{2}\right)}^{3}};C_{3}=-\frac{\left(1+\xi_{1}\right){\left(1+\xi_{2}\right)}^{2}\left(3\xi_{1}-\xi_{2}+2\right)}{{\left(\xi_{1}-\xi_{2}\right)}^{3}}\\ D_{3}=\frac{\left(1+\xi_{2}\right)\left(3\left(1+\xi_{1}\right)\left(\xi_{1}-\xi_{2}\right)+{\left(1+\xi_{2}\right)}^{2}\right)}{{\left(\xi_{1}-\xi_{2}\right)}^{3}} \end{array}\right.$$

(56) $$g_{1}\left(\xi_{1}\right)=\int_{0}^{\phi_{m}}\frac{d\phi }{\left(1+\xi_{1}-\cos\phi \right)}=\frac{2\arctan\left(\sqrt{(\xi_{1}+2)/\xi_{1}}\tan(\phi_{m}/2)\right)}{\sqrt{\xi_{1}(\xi_{1}+2)}}$$

(57) $$\begin{array}{ll} g_{2}\left(\xi_{2}\right)= & \int_{0}^{\phi_{m}}\frac{1}{{\left(1+\xi_{2}-\cos\phi \right)}^{3}}d\phi =\frac{(2\xi_{2}^{2}+4\xi_{2}+3)\arctan\left(\sqrt{(\xi_{2}+2)/\xi_{2}}\tan(\phi_{m}/2)\right)}{{\left(\xi_{2}(\xi_{2}+2)\right)}^{\left(5/2\right)}}+\\ & \tan(\phi_{m}/2)(1+\cos\phi_{m})\times \frac{4\xi_{2}^{2}+8\xi_{2}+3-3(1+\xi_{2})\cos\phi_{m}}{2\xi_{2}^{2}{\left(\xi_{2}+2\right)}^{2}{\left(1+\xi_{2}-\cos\phi_{m}\right)}^{2}} \end{array}$$

(58) $$\begin{array}{ll} g_{3}\left(\xi_{2}\right) & =\int_{0}^{\phi_{m}}\frac{\cos\phi }{{\left(1+\xi_{2}-\cos\phi \right)}^{3}}d\phi =\frac{3(\xi_{2}+1)\arctan\left(\sqrt{(\xi_{2}+2)/\xi_{2}}\tan(\phi_{m}/2)\right)}{{\left(\xi_{2}(\xi_{2}+2)\right)}^{\left(5/2\right)}}+\\ & \frac{{\left(\cos\phi_{m}+1\right)}^{2}\tan(\phi_{m}/2)}{4\xi^{2}{\left(\xi_{2}+2\right)}^{2}{\left(1+\xi_{2}-\cos\phi_{m}\right)}^{2}}\times (2\xi_{2}^{3}+5\xi_{2}^{2}+5\xi_{2}+\left(2\xi_{2}^{3}+7\xi_{2}^{2}+9\xi_{2}+6)\tan^{2}(\phi_{m}/2\right)) \end{array}$$

(59) $$\begin{array}{ll} g_{4}\left(\xi_{2}\right) & =\int_{0}^{\phi_{m}}\frac{\cos^{2}\phi }{{\left(1+\xi_{2}-\cos\phi \right)}^{3}}d\phi =\frac{(\xi_{2}^{2}+2\xi_{2}+3)\arctan\left(\sqrt{(\xi_{2}+2)/\xi_{2}}\tan(\phi_{m}/2)\right)}{{\left(\xi_{2}(\xi_{2}+2)\right)}^{\left(5/2\right)}}\\ & +\frac{\tan(\phi_{m}/2){\left(1+\cos\phi_{m}\right)}^{2}(\xi_{2}+1)}{4\xi_{2}^{2}{\left(\xi_{2}+2\right)}^{2}{\left(1+\xi_{2}-\cos\phi_{m}\right)}^{2}}\times \left(\xi_{2}^{2}+5\xi_{2}-(\xi_{2}^{2}-\xi_{2}-6)\tan^{2}(\phi_{m}/2)\right) \end{array}$$

(60) $$g_{5}\left(\xi_{1}\right)=\int_{0}^{\phi_{m}}\frac{1}{1+\xi_{1}-\cos\phi }\mathrm{dcos}\phi =\ln\left(\xi_{1}/(1+\xi_{1}-\cos\phi_{m})\right)$$

(61) $$g_{6}\left(\xi_{2}\right)=\int_{0}^{\phi_{m}}\frac{1}{{\left(1+\xi_{2}-\cos\phi \right)}^{3}}\mathrm{dcos}\phi =\frac{1}{2}\left(\frac{1}{{\left(1+\xi_{2}-\cos\phi_{m}\right)}^{2}}-\frac{1}{\xi_{2}^{2}}\right)$$

(62) $$g_{7}\left(\xi_{2}\right)=\int_{0}^{\phi_{m}}\frac{\cos\phi }{{\left(1+\xi_{2}-\cos\phi \right)}^{3}}\mathrm{dcos}\phi =-\frac{1}{2}\left(\frac{1+\xi_{2}-2\cos\phi_{m}}{{\left(1+\xi_{2}-\cos\phi_{m}\right)}^{2}}+\frac{1-\xi_{2}}{\xi_{2}^{2}}\right)$$

(63) $$\begin{array}{ll} g_{8}\left(\xi_{2}\right) & =\int_{0}^{\phi_{m}}\frac{\cos^{2}\phi }{{\left(1+\xi_{2}-\cos\phi \right)}^{3}}\mathrm{dcos}\phi \\ & =\frac{{\left(1+\xi_{2}\right)}^{2}}{2{\left(1+\xi_{2}-\cos\phi_{m}\right)}^{2}}-\frac{2\left(1+\xi_{2}\right)}{1+\xi_{2}-\cos\phi_{m}}+\frac{3\xi_{2}^{2}+2\xi_{2}-1}{2\xi_{2}^{2}}+\ln\left(\frac{\xi_{2}}{1+\xi_{2}-\cos\phi_{m}}\right) \end{array}$$

### 3.3. Special Case

When *ξ*_1_ = *ξ*_2_ = *ξ*, namely *t*_0_/*w*_0_ = *t*/*w*, these definite integrals from *I*_1_(*ξ*_1_, *ξ*_2_) to *I*_4_(*ξ*_1_, *ξ*_2_) defined in Equations (49)–(52) can be recalculated as:

(64) $$I_{1}\left(\xi \right)=I_{1}\left(\xi_{1},\xi_{2}\right)=\int_{0}^{\phi_{m}}\frac{\cos\phi d\phi }{{\left(1+\xi -\cos\phi \right)}^{2}}=\frac{2\arctan\left(\sqrt{(\xi +2)/\xi }\tan(\phi_{m}/2)\right)}{{\left(\xi (\xi +2)\right)}^{\left(3/2\right)}}+\frac{(1+\xi )\sin\phi_{m}}{\xi (\xi +2)(1+\xi -\cos\phi_{m})}$$

(65) $$\begin{array}{l} I_{2}\left(\xi \right)=I_{2}\left(\xi_{1},\xi_{2}\right)=\int_{0}^{\phi_{m}}\frac{\cos\phi d\phi }{{\left(1+\xi -\cos\phi \right)}^{4}}=\frac{(4\xi^{2}+8\xi +5)\arctan\left(\sqrt{(\xi +2)/\xi }\tan(\phi_{m}/2)\right)}{{\left(\xi (\xi +2)\right)}^{\left(7/2\right)}}+\\ \;\frac{{\left(1+\cos\phi_{m}\right)}^{3}}{8{\left(1+\xi -\cos\phi_{m}\right)}^{3}}\times \left(\begin{array}{l} \frac{\left(2\xi^{3}+8\xi^{2}+16\xi +11)\tan(\phi_{m}/2\right)}{\xi {\left(\xi +2\right)}^{3}}+\frac{4(3\xi^{3}+9\xi^{2}+16\xi +10)\tan^{3}(\phi_{m}/2)}{3\xi^{2}{\left(\xi +2\right)}^{2}}+\\ \frac{\left(2\xi^{3}+4\xi^{2}+8\xi +5)\tan^{5}(\phi_{m}/2\right)}{\xi^{3}(\xi +2)} \end{array}\right) \end{array}$$

(66) $$I_{3}\left(\xi \right)=I_{3}\left(\xi_{1},\xi_{2}\right)=\int_{0}^{\phi_{m}}\frac{\sin\phi \cos\phi d\phi }{{\left(1+\xi -\cos\phi \right)}^{4}}=\frac{\left(2+3\xi \right)}{6{\left(1+\xi \right)}^{2}\xi^{3}}-\frac{(3+3\xi -\cos\phi_{m})\cos^{2}\phi_{m}}{6{\left(1+\xi \right)}^{2}{\left(1+\xi -\cos\phi_{m}\right)}^{3}}$$

(67) $$\begin{array}{ll} I_{4}\left(\xi \right) & =I_{4}\left(\xi_{1},\xi_{2}\right)=\int_{0}^{\phi_{m}}\frac{\cos^{3}\phi d\phi }{{\left(1+\xi -\cos\phi \right)}^{4}}=\frac{(3\xi^{2}+6\xi +5)\arctan\left(\sqrt{(\xi +2)/\xi }\tan(\phi_{m}/2)\right)}{{\left(\xi (\xi +2)\right)}^{\left(7/2\right)}}+\\ & \frac{\left(1+\xi \right)\times \left(\begin{array}{l} 3\xi^{2}(2\xi^{2}+7\xi +11)\tan(\phi_{m}/2)+4\xi (\xi +2)(\xi^{2}+2\xi +10)\tan^{3}(\phi_{m}/2)\\ +3{\left(\xi +2\right)}^{2}(2\xi^{2}+\xi +5)\tan^{5}(\phi_{m}/2) \end{array}\right)}{3\xi^{3}{\left(\xi +2\right)}^{3}{\left(\xi +(\xi +2)\tan^{2}(\phi_{m}/2)\right)}^{3}} \end{array}$$

Until now, all the integral factors can be calculated by substituting Equations (49)–(63) selectively into Equations (38)–(48), or using directly Equations (64)–(67) when *ξ*_1_ = *ξ*_2_ = *ξ*, and then all the compliance elements of the flexure segments 1 and 4 can be also calculated by substituting these integral factors selectively into Equations (13)–(20) and Equations (28)–(31). Finally, the closed-form compliance matrix and precision factors of two-axis elliptical-arc-filleted flexure hinge can be obtained analytically by using Equations (1) and (37). Meanwhile, the two-axis elliptical-arc-filleted flexure hinge can also be further degenerated into other single-axis flexure hinges including the elliptical-arc-filleted (*a*_1_ → 0), the elliptical-arc (*a*_1_ → 0 and *l*_2_ = 0), the circular-arc-filleted (*a*_1_ → 0 and *a*_2_ = *b* = *r*), the circular (*a*_1_ → 0, *a*_2_ = *b* = *r* and *l*_2_ = 0), the elliptical-fillet (*a*_1_ → 0 and *φ*_m_ = π/2), the elliptical (*a*_1_ → 0, *φ*_m_ = π/2 and *l*_2_ = 0), the right-circular-fillet (*a*_1_ → 0, *φ*_m_ = π/2 and *a*_2_ = *b* = *r*), and the right-circular (*a*_1_ → 0, *φ*_m_ = π/2, *l*_2_ = 0 and *a*_2_ = *b* = *r*) flexure hinges [15,16,17,29,35]. Moreover, the closed-form compliance and precision matrices of two-axis flexure hinges with different cutout profiles defined in Figure 3b–f and the single-axis flexure hinges above can get directly in terms of the particular values of the geometric parameters of the two-axis elliptical-arc-filleted flexure hinge. In addition, the analytical compliance equations of two-axis elliptical-arc-filleted flexure hinge can be used for motion accuracy or maximum stress prediction, characteristic analysis, optimal design of the hinges itself, as well as can be employed in kinematic or static analysis, dimension optimization, workspace determination of flexure-based spatial compliant mechanisms. 

## 4. Validation and Numerical Simulation

Finite element analysis (FEA) is used to validate all analytical compliance equations in compliance and precision matrices derived in the previous section by utilizing the ANSYS Workbench software, and the effect of geometric parameters on compliance elements and hinge performance are discussed in this section.

### 4.1. Analytical Model

**Figure 9:**
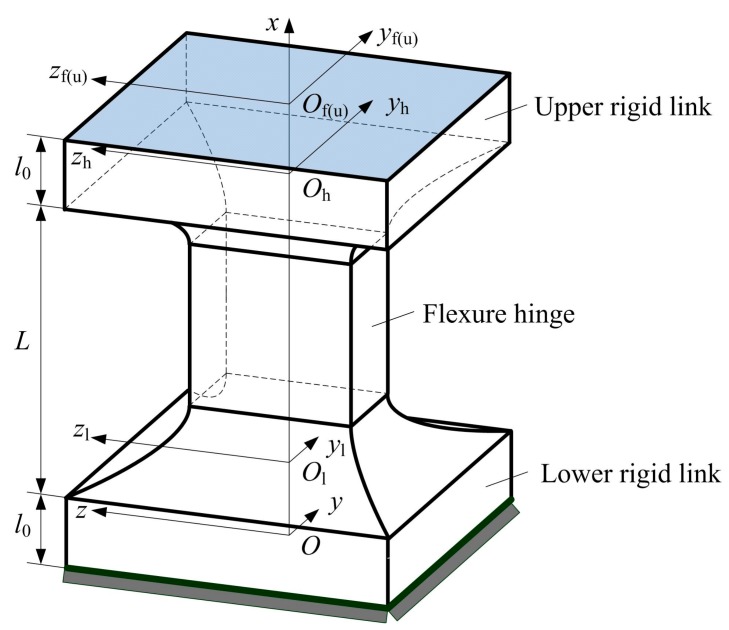
Geometric relationships among coordinate frames of two-axis elliptical-arc-filleted flexure hinge with two rigid links.

The FEA model of a flexure hinge includes the rigid links of both ends of the hinge besides the flexure hinge [18,19,20,21], namely the theoretical fixed end of the hinge will change and induce deformation in FEA model under the action of external load, and the FEA results of the hinge are that the rotations or deformations caused by the left-end section of the hinge were subtracted from the corresponding rotations or deformations caused by the right-end section of the hinge [25], thus there are different from the analytical model on the constraint conditions—see Figure 5 and the compliance calculations. Meanwhile, the point load is applied on a node or a test point [24,25], which is different from the actual applications of the hinge on the loading conditions. To overcome the above problems, an analytical model of two-axis elliptical-arc-filleted flexure hinge in accordance with the FEA model is proposed, and its geometric relationships among coordinate frames are shown in Figure 9, where the *O*-*xyz* and *O*_f_-*xy*_f_*z*_f_ are the global frame and the local frame established in the fixed end and the free end of the analytical model, respectively, the *O*_l_-*xy*_l_*z*_l_, *O*_h_-*xy*_h_*z*_h_ and *O*_u_-*xy*_u_*z*_u_ are the local frames established in the tips of lower rigid link, flexure hinge and upper rigid link, respectively, and *l*_0_ is the length of the rigid link.

Similar to Equation (1), the analytical compliance matrix of the two-axis elliptical-arc-filleted flexure hinge with two rigid links can be expressed as:

(68) $$C_{An}=J_{L}C_{L}J_{L}^{T}+J_{H}CJ_{H}^{T}+C_{U}$$

where ***C****_An_*, ***C****_L_*, ***C*** and ***C****_U_* indicate the compliance matrices of the analytical model, lower rigid link, two-axis elliptical-arc-filleted flexure hinge and upper rigid link, respectively, and each non-zero compliance element of ***C****_L_* and ***C****_U_* can be obtained by using *l*_0_, *w* and *t* instead of *l*_2_, *w*_0_ and *t*_0_ defined in Equation (6), respectively, ***J****_L_* and ***J****_H_* can be given by:

(69) $$J_{L}=\left(\begin{array}{cc} R_{L} & -R_{L}S_{L}(r_{L})\\ 0_{3\times 3} & R_{L} \end{array}\right);J_{H}=\left(\begin{array}{cc} R_{H} & -R_{H}S_{H}(r_{H})\\ 0_{3\times 3} & R_{H} \end{array}\right)$$

where both ***R****_L_* and ***R****_H_* are unit matrices here, and the position vectors ***r****_L_* and ***r****_H_* can be expressed as:

(70) $$r_{L}={\left(\begin{array}{ccc} l_{0}+L & 0 & 0 \end{array}\right)}^{T};r_{H}={\left(\begin{array}{ccc} l_{0} & 0 & 0 \end{array}\right)}^{T}$$

The length of the rigid link is short and its compliance is far less than the compliance of the hinge, as well as the deformation occurs mainly on the flexure hinge, thus the proposed analytical model can be used for verifying indirectly the correctness of the compliance and precision equations of two-axis elliptical-arc-filleted flexure hinge.

### 4.2. Finite Element Analysis and Validation

**Figure 10:**
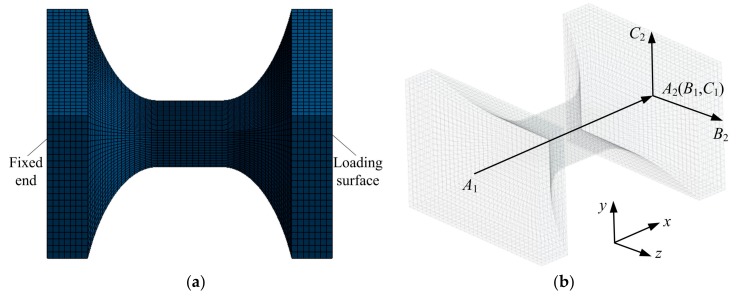
Two-axis elliptical-arc-filleted flexure hinge with two rigid links: (**a**) Finite element mesh model; (**b**) Path definitions.

The finite element mesh models of two-axis corner-filleted flexure hinges with two rigid links were generated by using twenty-node, three-dimensional hexahedral elements (Solid 186) with three translational degrees of freedom per-node, one of the hinge mesh models is shown in Figure 10a, of which its total nodes and total elements are 195,716 and 45,696, respectively. Meanwhile, the mesh-independent validation is carried out to eliminate the effect of mesh densities on the computational accuracy and reduce the calculation time. The one and the opposite end of each hinge model are fixed and loaded, respectively. In order to obtain or calculate better the compliance elements in FEA results, three different paths in finite element model are defined, as depicted in Figure 10b, where the *A*_1_-*A*_2_ is path 1, the *B*_1_-*B*_2_ is path 2, the *C*_1_-*C*_2_ is path 3, and *A*_1_-*A*_2_, *B*_1_-*B*_2_ and *C*_1_-*C*_2_ are parallel to *x*-, *z*- and *y*-axis, respectively, *A*_2_, *B*_1_ and *C*_1_ three points coincide, *A*_1_ and *A*_2_ are located on the geometric center of the fixed end and the loading surface, respectively, *B*_2_ and *C*_2_ are located on both sidelines of the loading surface, respectively, of which the output displacement of point *A*_2_ along the corresponding axis produced by unit force *f*_x_, *f_y_* or *f_z_* is used for obtaining the element *C_An_*_,11_, *C_An_*_,22_, or *C_An_*_,33_; the output displacements of point *B*_2_ along *x*-axis and point *A*_2_ along *z*-axis under unit moment *M_y_* are used for calculating the element *C_An_*_,55_ and obtaining the element *C_An_*_,35_, respectively; similarly, the output displacements of point *C*_2_ along *x*-axis and point *A*_2_ along *y*-axis under unit moment *M_z_* are used for calculating the element *C_An_*_,66_ and obtaining the element *C_An_*_,26_, respectively; the output displacement of point *B*_2_ along *y*-axis or point *C*_2_ along *z*-axis under unit moment *M_x_* is used for calculating the element *C_An_*_,44_. Six different two-axis corner-filleted flexure designs with two rigid links defined in Figure 3 are selected as the compliance comparisons between the analytical results and the finite element results. The geometric parameters of each model are listed in Table 1. For all design types, the linear elastic material properties are: the elastic modulus *E* = 2.0 × 10^11^ N/m^2^, Poisson’s ratio *v* = 0.3, the shear modulus *G* = 7.6923 × 10^10^ N/m^2^, and the density *ρ* = 8000 kg/m^3^. Notice, however, that only two-axis flexure hinges in small deformation are considered due to the stress concentration that occurs on the minimum cross section of the hinges, thus the static analysis with linear geometry setting is performed in the finite element model, which is different from the large deformation with non-linear geometry setting of the constant cross-section beams that are introduced by the material nonlinearity or the geometry nonlinearity [44,45], this is because the stress distributes uniformly on the constant cross-section beam. In addition, the nonlinear and the linear geometry settings in the finite element model of the hinges can get the same results in small deformation. Meanwhile, the analytical model is restricted to linear elastic materials and only applied to small deformation occasions.

Table 2 gives the analytical results and the finite element results, as well as the corresponding relative errors. It can be observed from Table 2 that the maximum relative error for two-axis elliptical-arc-filleted, circular-arc-filleted, elliptical-filleted, right-circular-filleted, hybrid elliptical-arc-filleted and circular-arc-filleted, and hybrid elliptical-filleted and right-circular-filleted flexure hinge between the analytical results and the finite element results is less than 10.5%, 6.5%, 9.0%, 7.5%, 6.5% and 8.0%, respectively, which shows that the analytical model predictions are in good conformity with the simulation results, and the analytical model can be also shown indirectly the exactness of the compliance and precision equations of two-axis corner-filleted flexure hinges deduced previously. Therefore, the analytical compliance and precision equations can be further used for predicting the effect of geometric parameters on compliance elements and performance of two-axis corner-filleted flexure hinges.

### 4.3. Compliance Ratio

To obtain the effect by joining the serially-connected flexure segments 2 and 3 on the compliance elements of two-axis flexure hinge with elliptical-arc notches, the analytical compliance equations is utilized, and the two-axis elliptical-arc-filleted flexure hinge is selected as an analysis example to compare with two-axis elliptical-arc flexure hinge by means of the following compliance ratio:

(71) $$r_{ij}=\frac{C_{ij,\mathrm{DEAF}}}{C_{ij,\mathrm{DEA}}}$$

where the subscript ‘’DEA’’ denotes the two-axis elliptical-arc flexure hinge defined by dual elliptical-arc cutout profiles.

**Figure 11:**
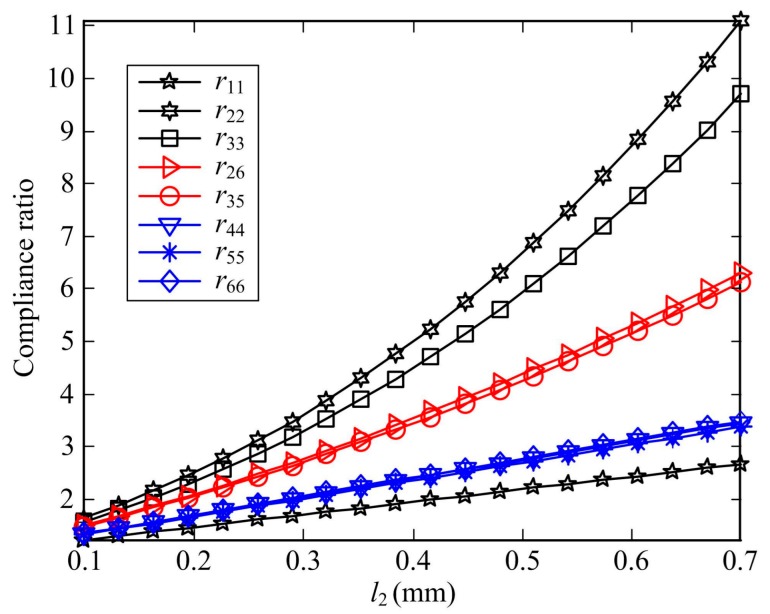
Compliance ratio of DEAF and DEA as a function of *l*_2_.

Figure 11 shows the variation of eight compliance ratios in terms of *l*_2_, the other geometric parameters listed in Table 1 except *l*_0_ = 0 and the material properties are all constant. According to the linear superposition principle, the compliance ratios *r*_11_, *r*_44_, *r*_55_, and *r*_66_ depend linearly on the length *l*_2_ and increase slowly. However, for the ratios *r*_22_, *r*_33_, *r*_26_ and *r*_35_, they depend non-linearly on the length *l*_2_, and the increased amplitude of *r*_22_ or *r*_33_ are more than *r*_26_ or *r*_35_. Meanwhile, it can be observed that the two-axis corner-filleted flexure hinges have higher capacity of rotation than two-axis flexure hinges when *l*_2_ is relatively large.

### 4.4. Compliance Precision Ratio

When designing a two-axis flexure hinge, we usually hope that the hinge can possess large compliance at the free end and relatively small off-axis compliance at the center of rotation to achieve high motion accuracy. Thus, the compliance and precision ratio can be an important performance index to evaluate the ability of preserving the center of rotation under the same displacement at the free end of the hinges [46]. For the transverse symmetric flexure hinges, according to the superposition principle, the axial compliance and precision ratio will be always equal to 2, which is not affected by the geometric parameters of the hinges. Hence, four compliance precision ratios can be defined as follows:

(72) $$r_{2}=\frac{C_{22}}{C_{Q,22}};r_{3}=\frac{C_{33}}{C_{Q,33}};r_{4}=\frac{C_{26}}{C_{Q,26}};r_{5}=\frac{C_{35}}{C_{Q,35}}$$

**Figure 12:**
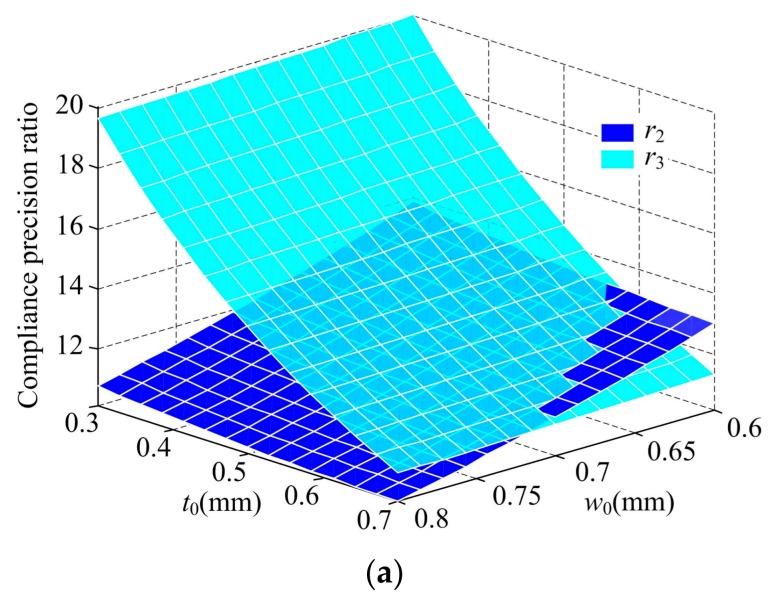

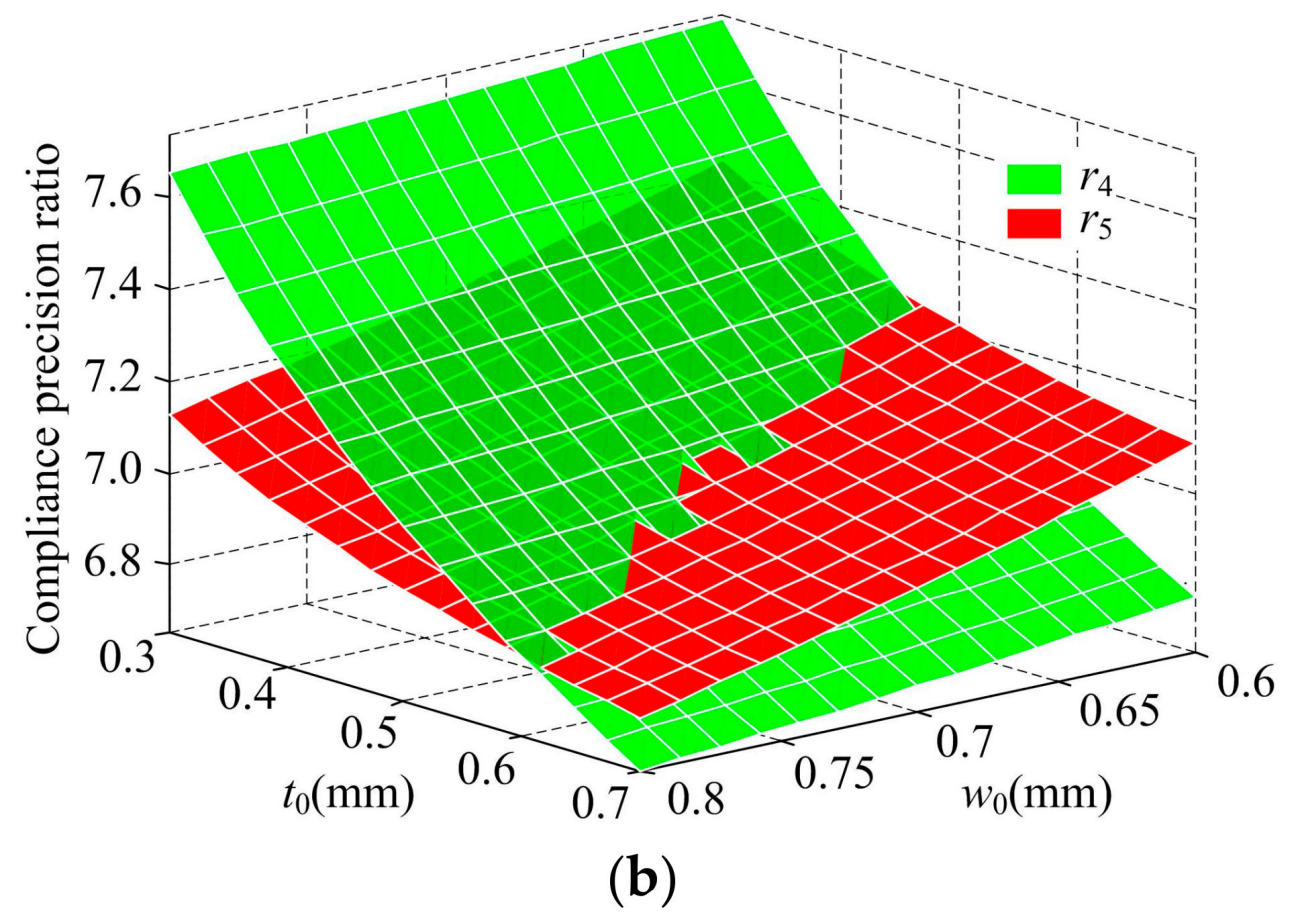
Compliance precision ratio of DEAF as a function of *t*_0_ and *w*_0_: (**a**) translational; (**b**) cross.

**Figure 13:**
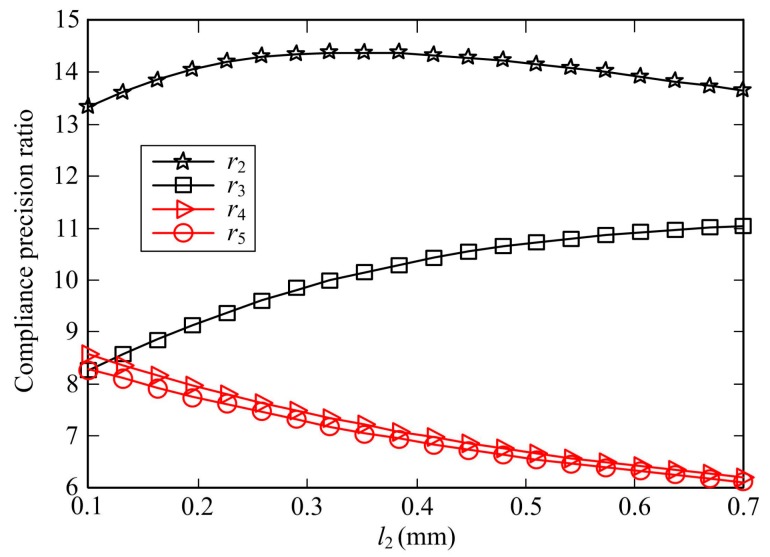
Compliance precision ratio of DEAF as a function of *l*_2_.

The larger compliance and precision ratios show that the flexure hinges have better ability and smaller relative motion error. Similarly, the two-axis elliptical-arc-filleted flexure hinge and its analytical compliance and precision equations are utilized to further investigate the effect of geometric parameters on the compliance precision ratio, all parameters listed in Table 1 except the corresponding variations and *l*_0_ = 0 keep constant. Figure 12 illustrates these trends that four compliance precision ratios of DEAF vary with the parameters *w*_0_ and *t*_0_, and Figure 13 illustrates the results of the compliance precision ratios as a function of *l*_2_.

From Figure 12a,b, the following conclusions regarding the two axis elliptical-arc-filleted flexure hinge but are also valid for other two-axis corner-filleted flexure hinges can be drawn:

- All the compliance precision ratios from *r*_2_ to *r*_5_ decrease when the parameters *w*_0_ and *t*_0_ increase, and vice versa; The ratios *r*_3_ and *r*_4_ are more sensitive to *t*_0_ than *w*_0_, and *w*_0_ has only slightly influence on them; The ratio *r*_2_ is less affected by the parameter *t*_0_ than *w*_0_; The effect of the parameters *w*_0_ and *t*_0_ on the ratio *r*_5_ are approximately the same.
- From the trends of the ratios *r*_2_ and *r*_3_, the ability of preserving the rotational center under pure force along *z*-axis is larger than the one along *y*-axis when *t*_0_ < *w*_0_, namely *r*_3_ > *r*_2_, and vice versa; but for the plots of the ratios *r*_4_ and *r*_5_, the ability of preserving the rotational center under pure moment about *z*-axis is larger than the one about *y*-axis when *ξ*_1_ > *ξ*_2_, namely *r*_4_ > *r*_5_, and vice versa.

The compliance precision ratios from *r*_2_ to *r*_5_ depend non-linearly on the parameter *l*_2_, as shown in Figure 13. The ability of preserving the rotational center under pure moment *M_y_* or *M_z_* decreases whereas the ability under pure force *f_z_* increases with increasing *l*_2_; but the ratio *r*_2_ increases firstly and then decreases slowly and can be achieved larger compliance precision ratio with respect to the other three; the ratio *r*_3_ is also larger than *r*_4_ and *r*_5_ as *l*_2_ increases, which show that the ability of preserving the rotational center under force is larger than the one under moment when the free end of two-axis elliptical-arc-filleted flexure hinge has same displacement and *l*_2_ is gradually larger.

## 5. Conclusions

This paper introduces a class of two-axis flexure hinges with different cutout proflies as an alternative of flexure hooke hinges used for compact, small flexure-based spatial compliant mechanisms. The compliance and precision matrices of a common two-axis elliptical-arc-filleted flexure hinge are formulated by using the Castigliano’s second theorem and the generic compliance modeling method, which can be used for other two-axis flexure hinges. FEA simulation is carried out to validate indirectly the analytical compliance and precision equations of six different types of two-axis corner-filleted flexure hinges, and the analytical results with respect to the simulation results are in good agreement. Besides, several non-dimensional functions are defined and formulated to analyze and study the performance of two-axis flexure hinges sensitivity to the geometric parameters.

## Figures and Tables

**Table 1:** Geometric parameters of two-axis corner-filleted flexure hinges with two rigid links.

	*t*_0_ (mm)	*w*_0_ (mm)	*a*_1_ (mm)	*a*_2_ (mm)	*b* (mm)	*l*_2_ (mm)	*φ*_m_	*l*_0_ (mm)	Type
1	0.5	0.8	2.0	1.5	1.0	0.4	60°	0.5	DEAF
2	0.5	0.8	1.0	1.0	1.0	0.4	60°	0.5	DCAF
3	0.7	1.0	2.0	2.5	1.5	0.5	90°	0.6	DEF
4	0.7	1.0	1.5	1.5	1.5	0.5	90°	0.6	DRCF
5	0.9	1.2	2.0	3.0	2.0	0.6	60°	0.7	HEACF
6	0.9	1.2	2.0	3.0	2.0	0.6	90°	0.7	HERCF

**Table 2:** Comparison of compliance elements between the analytical results and the FEA results (denoted by An and FEA, respectively).

		*C_An_*_,11_ (m/N)	*C_An_*_,22_ (m/N)	*C_An_*_,33_ (m/N)	*C_An_*_,26_/*C_An_*_,62_ (1/N)	*C_An_*_,35_/*C_An_*_,53_ (1/N)	*C_An_*_,44_ (rad/Nm)	*C_An_*_,55_ (rad/Nm)	*C_An_*_,66_ (rad/Nm)
1	An	2.135 × 10^−8^	2.780 × 10^−6^	1.149 × 10^−6^	1.449 × 10^−3^	−5.767 × 10^−4^	0.877	0.327	0.820
	FEA	2.289 × 10^−8^	2.968 × 10^−6^	1.268 × 10^−6^	1.541 × 10^−3^	−6.371 × 10^−4^	0.879	0.364	0.873
	Err	6.73%	6.33%	9.38%	5.97%	9.48%	0.23%	10.16%	6.07%
2	An	2.453 × 10^−8^	3.133 × 10^−6^	1.354 × 10^−6^	1.603 × 10^−3^	−6.627 × 10^−3^	0.981	0.375	0.908
	FEA	2.564 × 10^−8^	3.274 × 10^−6^	1.437 × 10^−6^	1.674 × 10^−3^	−7.057 × 10^−3^	0.976	0.401	0.948
	Err	4.33%	4.31%	5.78%	4.24%	6.09%	0.51%	6.48%	4.22%
3	An	1.647 × 10^−8^	2.296 × 10^−6^	1.231 × 10^−6^	8.228 × 10^−4^	−4.294 × 10^−4^	0.370	0.165	0.317
	FEA	1.746 × 10^−8^	2.459 × 10^−6^	1.341 × 10^−6^	8.789 × 10^−4^	−4.663 × 10^−4^	0.374	0.181	0.341
	Err	5.67%	6.63%	8.20%	6.38%	7.91%	1.07%	8.84%	7.04%
4	An	1.825 × 10^−8^	2.580 × 10^−6^	1.354 × 10^−6^	9.133 × 10^−4^	−4.667 × 10^−4^	0.408	0.180	0.351
	FEA	1.901 × 10^−8^	2.720 × 10^−6^	1.451 × 10^−6^	9.596 × 10^−4^	−4.993 × 10^−4^	0.411	0.194	0.371
	Err	4.00%	5.15%	6.69%	4.82%	6.53%	0.73%	7.22%	5.39%
5	An	1.421 × 10^−8^	1.606 × 10^−6^	1.012 × 10^−6^	4.844 × 10^−4^	−2.962 × 10^−4^	0.198	0.098	0.160
	FEA	1.496 × 10^−8^	1.707 × 10^−6^	1.081 × 10^−6^	5.147 × 10^−4^	−3.158 × 10^−4^	0.201	0.104	0.170
	Err	5.01%	5.92%	6.38%	5.89%	6.21%	1.49%	5.77%	5.88%
6	An	1.400 × 10^−8^	1.870 × 10^−6^	1.169 × 10^−6^	5.262 × 10^−4^	−3.209 × 10^−4^	0.198	0.097	0.160
	FEA	1.466 × 10^−8^	1.997 × 10^−6^	1.258 × 10^−6^	5.607 × 10^−4^	−3.441 × 10^−4^	0.201	0.105	0.171
	Err	4.50%	6.36%	7.07%	6.15%	6.74%	1.49%	7.62%	6.43%
